# Isoflurane Protects Against Human Endothelial Cell Apoptosis by Inducing Sphingosine Kinase-1 via ERK MAPK

**DOI:** 10.3390/ijms13010977

**Published:** 2012-01-17

**Authors:** Adnan M. Bakar, Sang Won Park, Mihwa Kim, H. Thomas Lee

**Affiliations:** 1 Department of Anesthesiology, College of Physicians and Surgeons of Columbia University, P&S Box 46 (PH-5), 630 West 168th Street, New York, NY 10032, USA; E-Mails: amb9045@nyp.org (A.M.B.); swp2109@columbia.edu (S.W.P.); mk2767@columbia.edu (M.K.); mk2429@columbia.edu (M.K.); 2 Department of Pediatrics, College of Physicians and Surgeons of Columbia University, CHN2-255, 3959 Broadway, New York, NY 10032, USA

**Keywords:** EA.hy926, sphingosine kinase, extracellular signal-regulated kinase, necrosis, volatile anesthetic

## Abstract

Endothelial dysfunction is a major clinical problem affecting virtually every patient requiring critical care. Volatile anesthetics are frequently used during the perioperative period and protect the heart and kidney against ischemia and reperfusion injury. We aimed to determine whether isoflurane, the most commonly used volatile anesthetic in the USA, protects against endothelial apoptosis and necrosis and the mechanisms involved in this protection. Human endothelial EA.hy926 cells were pretreated with isoflurane or carrier gas (95% room air + 5% CO_2_) then subjected to apoptosis with tumor necrosis factor-α or to necrosis with hydrogen peroxide. DNA laddering and *in situ* Terminal Deoxynucleotidyl Transferase Biotin-dUTP Nick-End Labeling (TUNEL) staining determined EA.hy926 cell apoptosis and percent LDH released determined necrosis. We also determined whether isoflurane modulates the expression and activity of sphingosine kinase-1 (SK1) and induces the phosphorylation of extracellular signal regulated kinase (ERK MAPK) as both enzymes are known to protect against cell death. Isoflurane pretreatment significantly decreased apoptosis in EA.hy926 cells as evidenced by reduced TUNEL staining and DNA laddering without affecting necrosis. Mechanistically, isoflurane induces the phosphorylation of ERK MAPK and increased SK1 expression and activity in EA.hy926 cells. Finally, selective blockade of SK1 (with SKI-II) or S1P_1_ receptor (with W146) abolished the anti-apoptotic effects of isoflurane. Taken together, we demonstrate that isoflurane, in addition to its potent analgesic and anesthetic properties, protects against endothelial apoptosis most likely via SK1 and ERK MAPK activation. Our findings have significant clinical implication for protection of endothelial cells during the perioperative period and patients requiring critical care.

## 1. Introduction

The integrity and viability of the vascular endothelium are essential for homeostasis of all vital organs. The endothelium provides a semi-permeable barrier for selective exchange of nutrients and fluids [1]. Furthermore, endothelial cells produce several critically important compounds including nitric oxide, sphingosine 1-phosphate (S1P) and prostaglandins, which not only regulate blood supply but also produce cytoprotection in many disease states including systemic inflammatory response syndrome and ischemia and reperfusion [1,2]. Clinically, endothelial dysfunction is a major problem in critical care medicine and during the perioperative period [3]. Furthermore, endothelial cell dysfunction plays a major role in many acute and chronic human diseases including ischemia and reperfusion injury, coronary artery disease, diabetes mellitus and systemic inflammatory response syndrome [1,3].

Volatile anesthetics are administered to virtually all patients subjected to general anesthesia and are an integral part of the perioperative period. Volatile anesthetics including isoflurane have non-anesthetic effects including effects on blood pressure, heart rate and systemic vascular resistance [4]. In addition, we previously reported that volatile anesthetics protected against renal ischemia reperfusion injury *in vivo* and had direct anti-inflammatory and anti-necrotic effects in cultured human kidney proximal tubule (HK-2) cells *in vitro* [5]. The initial anti-inflammatory mechanisms involve plasma membrane phosphatidylserine externalization with subsequent release of a potent anti-inflammatory cytokine TGF-β1 [5]. Furthermore, most volatile anesthetics are lipophilic molecules and have been shown to increase membrane fluidity and activate sphingomyelin hydrolysis [6]. The lysophospholipid S1P in particular is a product of sphingomyelin hydrolysis and functions as both an extracellular ligand for specific G protein coupled receptors as well as an intracellular second messenger in promoting cell growth, survival and reduction of apoptosis [7]. After inhalation, volatile anesthetics are first taken up by the circulatory system and endothelial cells are rapidly exposed; therefore the interactions between endothelial cells and volatile anesthetics are of great interest [8,9].

In this study we examined whether isoflurane reduces endothelial cell death due to necrosis or apoptosis and elucidated the mechanisms of isoflurane mediated endothelial cell protection. We test the hypothesis that isoflurane reduces endothelial apoptosis and necrosis via phosphorylation of extracellular signal-regulated kinase (ERK MAPK) and via induction of sphingosine kinase 1 (SK1) to increase production of a well characterized cytoprotective signaling molecule S1P [7,10].

## 2. Results and Discussion

### 2.1. Isoflurane Pretreatment Reduces Apoptosis in EA.hy926 Cells Exposed to TNF-α

**Figure 1:**
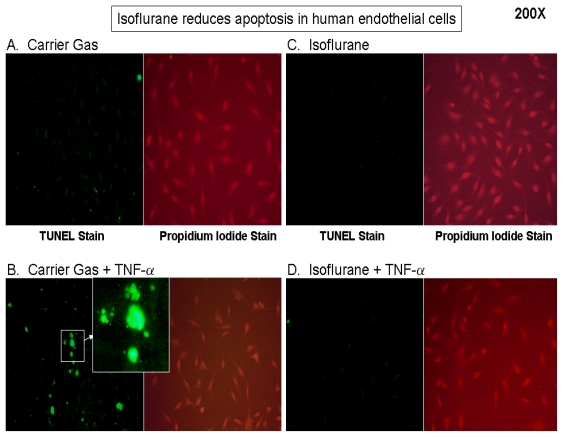

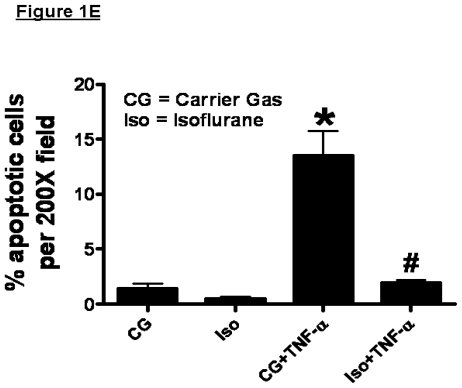
Representative TUNEL staining images (200×, representative of 4 independent experiments) demonstrate that isoflurane decreases EA.hy926 cell apoptosis induced by TNF-α. EA.hy926 cell were pretreated with either carrier gas (**A**,**B**) or isoflurane at 2.5% (2 MAC) isoflurane (**C**,**D**) for 16 hours. Subsequently, EA.hy926 cells were treated with either vehicle (saline, **A**,**C**) or with TNF-α 20 ng/mL for 48 hours (**B**,**D**). Enlarged image in **B** shows characteristic apoptotic cells with fragmented nuclei. The percentage of TUNEL positive cells were quantified as shown in **E**. *n* = 4 per group. * *p* < 0.05 *vs*. carrier gas group. ^#^ *p* < 0.05 *vs.* carrier gas + TNF-α. Data presented as mean ± SEM.

Human endothelial EA.hy926 cells exposed to carrier gas for 16 hours did not display any appreciable TUNEL staining (Figure 1A and 1E). Cells exposed to carrier gas for 16 hours followed by TNF-α 20 ng/mL for 48 hours showed significantly increased TUNEL positive cells (Figure 1B and 1E). EA.hy926 cells pretreated with isoflurane and then exposed to TNF-α 20 ng/mL for 48 hours showed ~6.7 fold (*p* < 0.05) reduction in TUNEL positive apoptotic cells when compared to cells exposed to carrier gas and TNF-α (Figure 1D and 1E).

**Figure 2:**
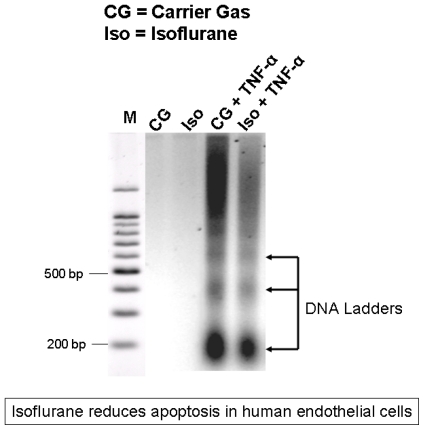
Representative DNA laddering images demonstrating reduced apoptosis in EA.hy926 cells with treatment with isoflurane (2.5% for 16 hours) followed by exposure to TNF-α (20 ng/mL for 48 hours) when compared to treatment with carrier gas followed by TNF-α. Representative of 4 independent experiments.

Eight hour isoflurane pretreatment also reduced the number of TUNEL positive apoptotic cells (data not shown). Similarly, EA.hy926 cells exposed to carrier gas for 8–16 hours did not display any appreciable DNA laddering. Cells exposed to carrier gas for 16 hours followed by TNF-α 20 ng/mL for 48 hours showed an increase in DNA laddering (Figure 2, representative of 4 independent experiments). Cells pretreated with isoflurane then exposed to TNF-α 20 ng/mL for 48 hours showed decreased DNA laddering when compared to cells exposed to carrier gas and TNF-α (Figure 2).

### 2.2. Isoflurane Does not Protect Against H_2_O_2_-Induced Necrosis in EA.hy926 Cells

After treatment with isoflurane or carrier gas, there were no significant differences in necrosis induced with 2 mM H_2_O_2_ between carrier gas (LDH released at 8 hours after 8 hours pretreatment = 29.4 ± 2.6%, *n* = 4) and isoflurane (LDH released at 8 hours after 8 hours pretreatment = 28.3 ± 2.5%, *n* = 4, *p* = 0.65) treated groups. Similarly, there were no significant differences at any other time points (2–12 hours, data not shown).

### 2.3. Isoflurane Treatment Phosphorylates ERK MAPK to Induce of SK1

**Figure 3:**
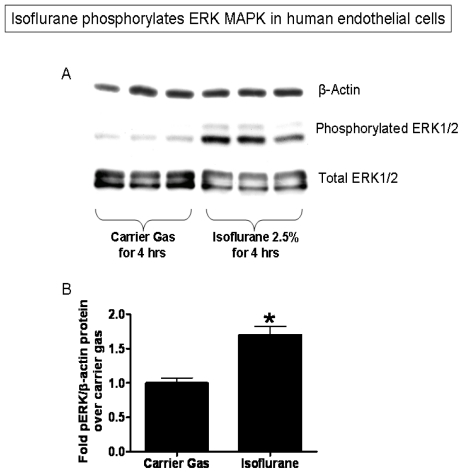
Isoflurane increases ERK MAPK phosphorylation in EA.hy926 cells without changing total ERK expression. (**A**) Representative immunoblotting images of β-actin, phosphorylated ERK and total ERK from 4 independent experiments; (**B**) Densitometric quantifications of band intensities relative to beta-actin. *n* = 4 per group. * *p* < 0.05 *vs*. carrier gas group. Cells were treated with either carrier gas or with 2.5% isoflurane in carrier gas for 4 hours.

EA.hy926 cells pretreated with isoflurane (2.5% or 2 MAC) for 2–4 hours show increased phosphorylation of ERK MAPK when compared to EA.hy926 cells exposed to carrier gas (Figure 3, 4 hour pretreatment with isoflurane shown).

**Figure 4:**
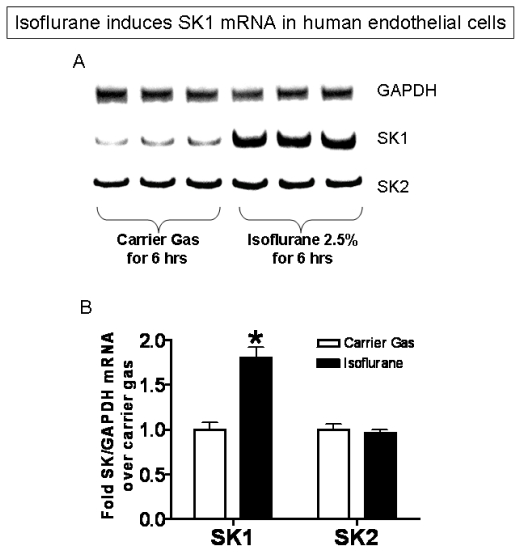
Isoflurane treatment selectively increases sphingosine kinase-1 (SK1) mRNA expression in EA.hy926 cells. (**A**) Representative RT-PCR images for GAPDH, SK1 and SK2 from 4 independent experiments; (**B**) Densitometric quantifications of band intensities for SK1 and SK2 relative to GAPDH from RTPCR reactions. *n* = 4 per group. * *p* < 0.05 *vs*. carrier gas group. Cells were exposed to either carrier gas or isoflurane 2.5% (2 MAC) for 6 hours.

**Figure 5:**
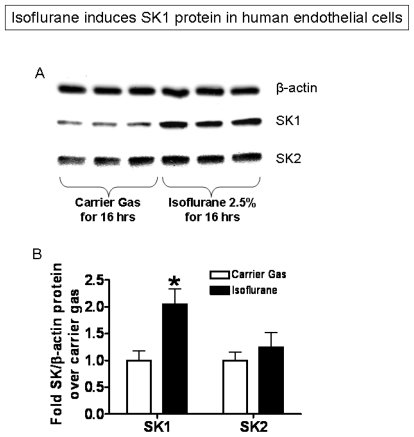
Isoflurane treatment selectively increases sphingosine kinase-1 (SK1) protein expression in EA.hy926 cells. (**A**) Representative immunoblotting images for β-actin, SK1 and SK2 from 4 independent experiments; (**B**) Densitometric quantifications of band intensities relative to β-actin. *n* = 4 per group. * *p* < 0.05 *vs*. carrier gas group. Cells were exposed to either carrier gas or isoflurane 2.5% or 2 MAC for 16 hours.

In addition, EA.hy926 cells exposed to isoflurane (2.5% or 2 MAC) for 3–6 hours show increased SK1 mRNA when compared to cells exposed to carrier gas (Figure 4, 6 hour exposure shown). Cells treated with isoflurane also showed an increase in SK1 protein expression (Figure 5, 16 hour exposure shown). In contrast to SK1, treatment of cells with isoflurane did not increase mRNA or protein expression for SK2 (Figure 4 and Figure 5).

**Figure 6:**
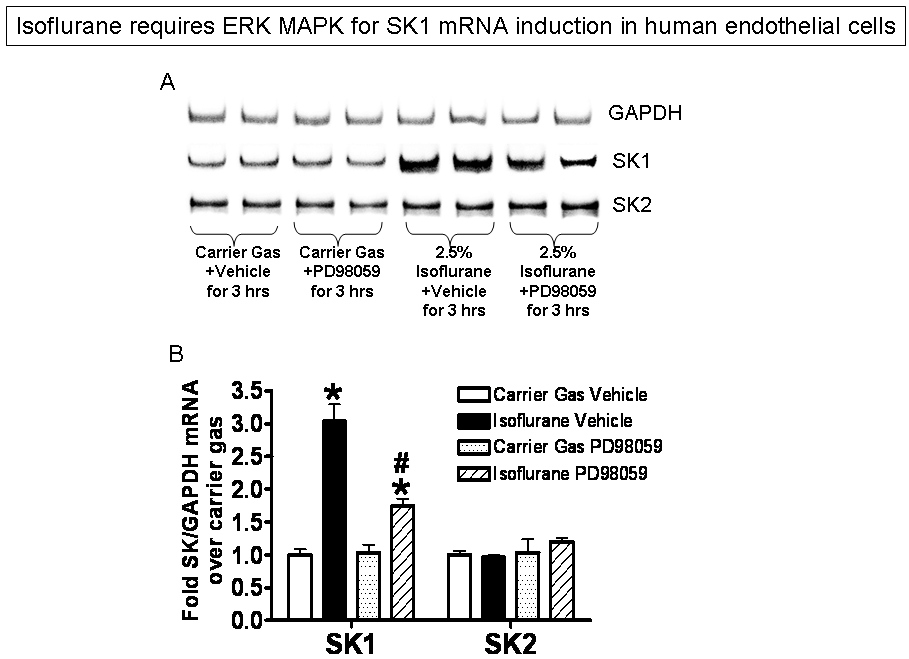
Inhibition of ERK MAPK phosphorylation with PD98059 (a selective MEK1 inhibitor) significantly reduced isoflurane-mediated induction of SK1 mRNA in EA.hy926 human endothelial cells. (**A**) Representative RT-PCR images for GAPDH, SK1 and SK2 from 4 independent experiments; (**B**) Densitometric quantifications of band intensities for SK1 and SK2 relative to GAPDH from RTPCR reactions. *n* = 4 per group. * *p* < 0.05 *vs*. carrier gas group. ^#^ *p* < 0.05 vs isoflurane+vehicle group. Cells were exposed to either carrier gas or isoflurane 2.5% (2 MAC) for 3 hours.

Finally, inhibition of ERK MAPK phosphorylation with a selective MEK1 inhibitor (PD98059) significantly reduced isoflurane-mediated (2.5% for 3 hours) induction of SK1 mRNA in EA.hy926 human endothelial cells (Figure 6).

### 2.4. SK or S1P_1_ Receptor Inhibition Prevents Isoflurane-Mediated Reduction in Apoptosis

**Figure 7:**
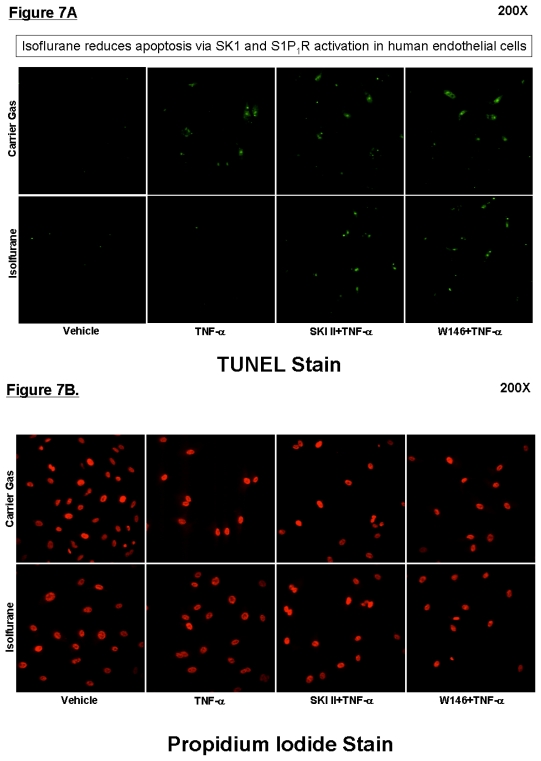
Inhibition of sphingosine kinase prevents isoflurane-mediated reduction in apoptosis. Representative TUNEL staining images (200×, representative of 4 independent experiments) demonstrate that SKI-II, a potent SK inhibitor, or W146, a selective S1P_1_ receptor antagonist, attenuates isoflurane-mediated reduction in TUNEL positive apoptotic cells. EA.hy926 cells were pretreated with 2 MAC isoflurane for 8 hours and apoptosis induced by 8 hour treatment with 20 ng/mL TNF-α. Propidium iodide nuclear stain was also performed to demonstrate equivalent cell number among experimental groups.

Figure 7 (TUNEL staining) shows that a selective SK inhibitor (SKI-II) or a selective S1P_1_ receptor antagonist (W146) reversed the protective effects of isoflurane (2.5% for 8 hours) against TNF-α induced apoptosis (induced with 24 hours of 20 ng/mL TNF-α treatment). Propidium iodide nuclear stain (lower panel) was performed to demonstrate equivalent cell number among experimental groups. DNA laddering assay also showed that SKI-II blocked the anti-apoptotic effects of isoflurane treatment (data not shown).

### 2.5. Isoflurane Increases SK Activity and S1P Formation in EA.hy926 Cells

Isoflurane treated EA.hy926 cells had demonstrated 1.27 + 0.07 fold higher SK activity compared to carrier gas treated cells (*n* = 4, *p* < 0.01). HPLC analysis for S1P showed increased S1P synthesis in EA.hy926 cells treated with isoflurane (1.27 + 0.04 fold, *n* = 6, *p* < 0.001) compared to the carrier gas treated group.

### 2.6. Discussion

Volatile anesthetics are one of the most widely used drugs in the USA during the perioperative period. In addition to its analgesic and anesthetic properties, they are well known to have powerful non-anesthetic properties. For example, several of the clinically utilized volatile anesthetics have negative inotropic effects, decrease systemic vascular resistance and venous return [4]. Furthermore, volatile anesthetics have cytoprotective properties in several cell types. For example, several clinically utilized volatile anesthetics including isoflurane precondition the myocardium against ischemia and reperfusion injury [11]. We have previously demonstrated that isoflurane as well as sevoflurane produces powerful anti-necrotic and anti-inflammatory effects in renal proximal tubule cells *in vivo* and *in vitro* [5,12,13]. Harnessing these non-anesthetic properties of volatile anesthetics may have important clinical implications for critically ill patients anesthetized in the OR and sedated in the ICU.

We determined whether isoflurane reduces apoptosis or necrosis in EA.hy926 cells. Apoptosis and necrosis are two major pathways of cell death [14]. Apoptosis occurs in a pre-programmed and energy dependent manner involving activation of several cysteine proteases called caspases. These caspases initiate a complex cascade of events which lead to DNA fragmentation and cell death. Necrosis, however, is thought to be a passive, non-energy dependent process characterized by rapid cell swelling (oncosis), mitochondrial changes and eventual cell lysis with release of the cytoplasmic contents into the interstitial surroundings causing the recruitment of inflammatory cells [14].

In this study, we show that isoflurane selectively inhibited TNF-α induced apoptosis in EA.hy926 cells. Previous studies demonstrated volatile anesthetic-mediated protection against endothelial cell death although the exact mechanisms of protection were not fully elucidated. Isoflurane has been shown to decrease ICAM-1, VCAM and NF-κB expression [15] and reduces cytokine-induced apoptosis via protein kinase C activation [8] as well as by modulating mitochondrial ATP-sensitive potassium channels [16]. We demonstrate lack of protection against necrosis in EA.hy926 cells. This is in contrast to previous studies that showed anti-necrotic effects of isoflurane in renal epithelial cells [13] and in aortic endothelial cells [9]. The effects of isoflurane may be tissue specific as isoflurane reduced cell death in aortic endothelial cells but had no effect on pulmonary artery endothelial cells after H_2_O_2_ treatment [9]. Taken together, isoflurane most likely modulates diverse anti-apoptotic and anti-necrotic signaling pathways in a tissue and cell death specific manner.

One of the limitations of our model of necrosis is the concentration of H_2_O_2_ used (2 mM). Endothelial cell would not likely encounter such a massive stress *in vivo.* H_2_O_2_ and its metabolite, the hydroxyl radical, contribute to the pathogenesis of reperfusion injury in many organ systems and cell types [17]. H_2_O_2_ is widely used *in vitro* as it easily penetrates the cell membrane and causes intracellular injury. Our preliminary studies showed that H_2_O_2_-induced necrosis in *EA.hy926 cells* is dose- and time-dependent. We chose to utilize 2 mM H_2_O_2_ as preliminary experiments demonstrated that these doses killed endothelial cells rapidly (within 4–8 hours). Lower doses (~0.5–2 mM) killed cells more slowly (>6 hours). Previous studies, including the ones from our laboratory, suggest immortalized cells *in vitro* require higher concentrations of oxidant stress for induction of necrosis [17].

In this study, we demonstrate that isoflurane induces phosphorylation of ERK MAPK and induces SK1 mRNA, protein and activity in human endothelial cells. We propose that activation of endothelial SK1 and ERK MAPK mediates the anti-apoptotic effects of isoflurane against TNF-α induced apoptosis in EA.hy926 cells. We previously showed that isoflurane externalized plasma membrane phosphatidylserine with subsequent release of TGF-β1 [5]. We also demonstrated that volatile anesthetics phosphorylated ERK MAPK via TGF-β1 signaling in human proximal tubule epithelial cells and this ERK MAPK activation was directly responsible for the protective effects of volatile anesthetics in the kidney [5]. Furthermore, we previously showed that isoflurane induced expression and activity of SK1 in human proximal tubule cells [13] as well as human intestinal epithelial cells [18] that was directly responsible for isoflurane-mediated kidney and intestine protection, respectively. In above studies, blockade of SK1 with a selective inhibitor (SKI-II, 4-[[4-(4-Chlorophenyl)-2- thiazolyl]amino]phenol) or deletion of SK1 (with SK1 KO mice) abolished the protective effects of isoflurane treatment [12]. Other studies have implicated the upregulation of S1P by isoflurane as the mode of protection from hypoxic-ischemic brain injury in rats [10].

Volatile anesthetics are powerful modulators of plasma membrane lipid biochemistry including sphingolipid metabolism [6]. Once administered, volatile anesthetics rapidly interact with vascular endothelium, which is a potent contributor of plasma S1P [19]. Sphingolipids and their metabolites have been shown to play roles in various physiologic and pathophysiologic pathways [20]. SK has two mammalian isoforms, SK1 and SK2, which catalyze the ATP-dependant phosphorylation of sphingosine to S1P [21]. SK1 is found in the cytosol of eukaryotic cells, and when activated translocates to the plasma membrane. SK2 is found in the nucleus and endoplasmic reticulum and its function is less well known. The phosphorylation product of sphingosine kinase, S1P, promotes cell growth and differentiation and is a powerful anti-apoptotic ligand for specific G protein coupled S1P receptors [7,22]. It is well documented that S1P produces anti-apoptotic effects in various cell types [13,23,24]. Activation of S1P receptors has been shown to protect against ischemia reperfusion injury in the heart [25], liver [26] and kidney [27]. Furthermore, ERK MAPK is a component of the MEK1/MAPK/Ras pathway which governs proliferation, differentiation, and cell survival [28]. Indeed, SK-1 and S1P has been shown to upregulate the ERK MAPK pathway as the mechanism for increasing cell survival [29].

While several previous studies demonstrate tissue-protective effects of several clinically utilized volatile anesthetics [11,12,25,29,30], there are studies that show detrimental and cytotoxic effects of volatile anesthetics. In particular, volatile anesthetics have been shown to produce detrimental neuronapoptotic effects in the developing brain of rats and mice [31,32]. Volatile anesthetics reduce dendritic branching and synaptogenesis [33–35], cause apoptotic neuron-degeneration in the developing hippocampus and neocortex [32] and cognitive dysfunction in rats [36]. It is hard to reconcile the differences of these findings to our study. Perhaps short term exposure to volatile anesthetics in an adult cell line results in cytoprotection whereas relative long exposure in neonatal tissue may produce detrimental effects. Indeed, there are studies that demonstrate neuroprotective effects of clinically used volatile anesthetics. In the setting of cerebral ischemia, isoflurane pretreatment improves cerebral blood flow [37] and benefits both short and long neurological outcome [38] in rats. It has also been shown to be neuroprotective in the setting of traumatic brain injury [39,40].

## 3. Experimental Section

### 3.1. Cell Culture

Immortalized human umbilical vein endothelial cell line (EA.hy926, purchased from American Type Culture Collection, Manassas, VA, USA) were grown and passaged in culture medium (Dulbecco’s modified Eagle’s medium + 10% fetal bovine serum) at 37 °C in a 95% room air + 5% CO_2_ humidified environment. This cell line has been characterized extensively and retains physiologic and functional characteristics of endothelial cells [41].

### 3.2. Exposure of EA.hy926 Cells to Isoflurane

EA.hy926 cells were exposed to isoflurane (2-Chloro-2-(difluoromethoxy)-1,1,1-trifluoro-ethane, Abbott Laboratories, Chicago, IL, USA) as described previously [5]. In brief, EA.hy926 cells were placed in an air-tight, 37 °C, humidified modular incubator chamber (Billups-Rothenberg, Del Mar, CA, USA) and exposed to 2.5% (2 MAC) isoflurane (where 1 MAC is defined as the percent concentration in the alveolus of an inhaled anesthetic agent required to prevent 50% of subjects from moving in response to a painful stimulus when used as the sole anesthetic) mixed with 95% air + 5% CO_2_ (carrier gas). Exposure to isoflurane was between 4 to 16 hours. We have previously demonstrated that clinically relevant concentrations of volatile anesthetics (0.5–2 MAC) produce cytoprotective signaling in human proximal tubule (HK2) cells [5], and used a similar protocol. Control cells were exposed to carrier gas in an identical modular incubator for the same duration.

### 3.3. Induction of EA.hy926 Cell Necrosis with Hydrogen Peroxide (H_2_O_2_)

After pretreatment (16 hours) with isoflurane or with carrier gas, EA.hy926 cells were exposed to oxidative stress necrosis with 2 mM H_2_O_2_ in the 37 °C modular incubator exposed to 95% air + 5% CO_2_ for 2–24 hours. After exposure, culture media was collected and assayed for lactate dehydrogenase (LDH) released from necrotic cells. We previously demonstrated that this dose of H_2_O_2_ causes significant necrosis without appreciable apoptosis in cell culture [5].

### 3.4. Induction of EA.hy926 Cell Apoptosis Using Tumor Necrosis Factor-α (TNF-α)

To induce apoptosis, EA.hy926 cells were exposed to TNF-α (20 ng/mL) in the 37 °C modular incubator exposed to 95% air + 5% CO_2_ for 48 hours after 16 hours of pretreatment with 2 MAC isoflurane or with carrier gas. This dose was determined in preliminary experiments to produce consistent inflammatory changes in cell culture (data not shown).

### 3.5. Detection of Apoptosis

We used 2 independent techniques to detect apoptosis in EA.hy926 cells 1) with *in situ* Terminal Deoxynucleotidyl Transferase Biotin-dUTP Nick-End Labeling (TUNEL) Assay and 2) with DNA laddering assay*. In situ* labeling of fragmented DNA was performed with TUNEL (green fluorescence) with a commercially available *in situ* cell death detection kit (Roche, Nutley, NJ, USA) according to the manufacturer’s instructions. To visualize the total number of cells in the field, slides were also stained with propidium iodide (red fluorescence). The slides were evaluated blindly through the counting of the labeled cells in 200× magnified fields.

For DNA laddering assay, DNA fragments isolated from EA.hy926 cells were extracted according to the methods of Herrmann *et al.* [42] and was electrophoresed at 70 V in a 2.0% agarose gel in Tris-acetate-EDTA buffer. This method of DNA extraction selectively isolates apoptotic, fragmented DNA and leaves behind majority of intact chromatin. The gel was stained with ethidium bromide and photographed under UV illumination. DNA ladder markers (100 bp) were added to a lane of each gel as a reference for the analysis of internucleosomal DNA fragmentation.

### 3.6. Detection of necrosis with LDH Measurement

LDH released into cell culture media as an indicator of rapid cell lysis from necrosis was measured with an LDH assay kit (Promega, Madison, WI, USA). LDH released into the media is expressed as percentage of total cellular LDH measured after cells are lysed with 1% Triton X-100.

### 3.7. Immunoblot Analysis

After exposure to carrier gas or isoflurane, EA.hy926 cells were probed for SK1, sphingosine kinase-2 (SK2) and phosphorylated or total ERK MAPK by immunoblot analysis as previously described [43]. In brief, EA.hy926 cells were lysed with ice cold RIPA buffer [150 mM NaCl, 50 mM Tris-HCl, 1 mM EDTA, and 1% Triton-X (pH 7.4)] with phosphatase and protease inhibitors. Ten to 50 μg of EA.hy926 lysates were electrophoresed on a 10% polyacrylamide gel and transferred to a PVDF membrane. Primary antibodies for SK1 (1:1000 dilution) were purchased from Cell Signaling Technologies (Danvers, MA, USA). Primary antibodies for SK2 (1:1000) were purchased from Abcam (Cambridge, MA, USA). Primary antibodies for β-actin (1:5000) were purchased from Sigma (St. Louis, MO, USA). Primary antibodies for phosphorylated ERK (1:1000) and total ERK MAPK (1:1000) were purchased from Santa Cruz biotechnology (Santa Cruz, CA, USA). As phosphorylation products are synthesized quicker, when blotting for ERK MAPK isoflurane exposure was limited to 4 hours. We have previously determined that 16 hour volatile anesthetic exposure was optimal to capture sphingosine kinase expression. Secondary antibody [sheep anti-mouse or donkey anti-rabbit igG conjugated to horseradish peroxidase at 1:5000 dilution (GE Healthcare, Piscataway, NY, USA)] was detected with enhanced chemiluminescence immunoblotting detection reagents (Amersham, Piscataway, NJ, USA) with subsequent exposure to a CCD camera coupled to a UVP Bioimaging System (Upland, CA, USA) and a personal computer.

### 3.8. Semi-Quantitative Reverse Transcription–Polymerase Chain Reaction (RTPCR)

After exposure to carrier gas or isoflurane, we detected mRNA expression of SK1 and SK2 as previously described using the Access RT-PCR System (Promega) [43]. The quantitative accuracy of our RT-PCR technique was first confirmed for each primer pair used. The PCR cycle number for each primer pair was optimized for linear increases in densitometric band intensity measurements with increasing PCR cycles from 15–26. The starting amount of RNA (0.25–1 μg) was also optimized for linear increase in densitometric band intensity measurements at an optimized cycle of PCR. SK1 and SK2 primers were designed based on published GenBank sequences for human (Table 1) and to amplify a genomic region that spans one or more introns to distinguish mRNA products from genomic DNA contamination. Semiquantitative RT-PCR was done under conditions yielding linear results for GAPDH to confirm equal RNA input. Five microliters of the RT-PCR product were analyzed on a 6% acrylamide gel stained with SYBR green (Invitrogen, Carlsbad, CA, USA) for analysis with a UVP Bioimaging System.

### 3.9. SK Activity Assay

SK activity was measured as described by Vessey *et al.* [44] with some modifications as described [12].

### 3.10. High-Pressure Liquid Chromatography (HPLC) Detection of S1P

Confluent EA.hy926 cells were sonicated in PBS (pH 7.2). Aliquots were used for protein assay and the remainder of EA.hy926 cellular lysates were processed by HPLC to measure S1P levels as described by Min *et al.* [45] with two steps of sample pretreatment: enzymatic dephosphorylation of S1P by alkaline phosphatase (100 U/sample, Sigma) and subsequent analysis of ophthalaldehyde derivatives of the liberated sphingosine bases by HPLC. By introducing C17 S1P (Avanti Polar Lipids, Inc., Alabaster, AL) as an internal standard, S1P present in a sample can be quantified on a C18 reversed-phase column with a simple mobile phase of acetonitrile:deionized distilled water (89:11, v/v) and expressed as fold increase over the carrier gas-treated group.

### 3.11. Statistical Analysis

The data were analyzed with t-test when means between two groups were compared or with one-way ANOVA plus Tukey post hoc multiple comparison test to compare mean values across multiple treatment groups. In all cases, *p* < 0.05 was taken to indicate significance. All data are expressed as mean ± SEM.

### 3.12. Reagents and Protein Determination

Unless otherwise specified, all chemicals were obtained from Sigma. Protein contents were determined with a bicinchoninic acid protein assay kit (Pierce Chemical Co., Rockford, IL), using bovine serum albumin as a standard.

## 4. Conclusion

We demonstrate in this study that a clinically used volatile anesthetic (isoflurane) reduces apoptosis in human endothelial cells, presumably via ERK MAPK phosphorylation, SK1 induction and increased S1P synthesis. *In vivo* studies will be required to translate our *in vitro* findings. Further elucidation of the mechanisms of protection may lead to advancements in the treatment of diverse human diseases involving endothelial dysfunction.

## Figures and Tables

**Table 1:** Primers used to amplify mRNAs encoding murine SK1, SK2, and GAPDH based on published GenBank sequences for mice.

Primers	Accession Number	Sequence (Sense/Antisense	Product Size (bp)	Cycle Number	Annealing Temp. (°C)
Human SK1	NM_021972	5′-ATCTCCTTCACGCTGATGC-3′ 5′-GTGCAGAGACAGCAGGTTCA-3′	330	26	66
Human SK2	NM_020126	5′-GGAGGAAGCTGTGAAGATGC-3′ 5′-GCAGGTCAGACACAGAACGA-3′	482	22	66
Human GAPDH	NM_002046	5′-ACCACAGTCCATGCCATCAC-3′ 5′-CACCACCCTGTTGCTGTAGCC-3′	450	15	65

SK = sphingosine kinase; GAPDH = glyceraldehyde 3-phosphate dehydrogenase; Respective anticipated RT-PCR product size, PCR cycle number for linear amplification and annealing temperatures used for each primer are also provided.
